# Supplementary material for: Neonatal genetics of gene expression reveal potential origins of autoimmune and allergic disease risk
Source: Nat Commun. 2020 Jul 28;11:3761. doi: 10.1038/s41467-020-17477-x (PMC7387553; doi:10.1038/s41467-020-17477-x)
Supplement: Supplementary file 1 — Supplementary Information [file 41467_2020_17477_MOESM1_ESM.pdf]

Supplementary Information

Supplementary Table 1–3, Supplementary Figure 1–16

Neonatal genetics of gene expression reveal potential origins of autoimmune and allergic disease risk

Huang Q.Q. *et al*.

Supplementary Table 1: Number of *cis*-eGenes and independent *cis*-eQTLs.

Cell type	Treatment	Sample size	#eGenes	#eGene-eSNP associations	#eGenes with multiple independent eQTLs (proportion)	#Independent eQTL signals
Myeloid cells	Resting	116	136	9462	1 (0.7%)	137
	LPS	125	376	25110	12 (3.2%)	386
T cells	Resting	126	971	68161	53 (5.5%)	1027
	PHA	127	1347	100091	85 (6.3%)	1436

The number of independent eQTL signals were obtained from the conditional analysis.

Supplementary Table 2: *Trans*-associations that are mediated by *cis*-eGenes.

Condition	eSNP	*Cis*-eGene	*Trans*-eGene	Total Effect	a	b	Mediation Effect (%)	P-value	FDR
Resting M	12:56435929	*RPS26*	*MYH10*	-1.00	-1.23	0.62	-0.77 (77.0%)	<0.001	<0.001
LPS M	12:56435929	*RPS26*	*MYH10*	-0.94	-1.10	0.22	-0.24 (25.7%)	0.015	0.035
Resting T	12:56435929	*RPS26*	*MYH10*	-1.30	-1.38	0.20	-0.28 (21.5%)	0.006	0.026
Resting T	12:56435929	*RPS26*	*MIR130A*	-1.10	-1.38	0.26	-0.36 (32.5%)	0.019	0.039
Resting T	12:56435929	*RPS26*	*STX1B*	-1.13	-1.38	0.25	-0.35 (30.8%)	0.042	0.066
Resting T	12:56389293	*RPS26*	*MIR1471*	0.79	1.28	0.02	0.02 (3.0%)	0.920	0.949
Resting T	12:56435929	*RPS26*	*IP6K2*	-0.92	-1.38	0.36	-0.50 (54.2%)	0.009	0.032
Resting T	12:56444632	*SUOX*	*MYH10*	1.05	-0.47	-0.06	0.03 (2.7%)	0.506	0.643
PHA T	12:56444632	*SUOX*	*MYH10*	0.58	-0.56	0.13	-0.07 (-12.3%)	0.060	0.083
Resting T	12:56444632	*SUOX*	*MIR130A*	0.93	-0.47	0.02	-0.01 (-1.2%)	0.949	0.949
PHA T	12:56435412	*SUOX*	*MIR130A*	0.46	-0.56	0.13	-0.07 (-15.8%)	0.036	0.063
Resting T	12:56444632	*SUOX*	*STX1B*	0.92	-0.47	0.07	-0.03 (-3.5%)	0.702	0.819
Resting T	4:119170256	*SNHG8*	*MIR330*	-1.03	-1.33	0.49	-0.65 (62.8%)	0.001	0.008
PHA T	4:119170256	*SNHG8*	*MIR330*	-1.16	-1.25	0.42	-0.53 (45.7%)	0.014	0.035

Rows in the table are the 14 mediation trios that were tested, with not significant ones (FDR >0.05) in grey. The "Condition" column indicates four groups, with "M" and "T" indicating myeloid cells and T cells, respectively, which were stimulated by either lipopolysaccharide (LPS) or phytohemagglutinin (PHA). The "eSNP" column indicates the lead SNP in each *trans* locus that are also associated with local genes ("*Cis*-eGene"). The statistics were obtained from the bootstrap method. The column "Total Effect" shows the total effect of the eQTL on the *trans*-eGene; "a" indicates the effects of the eQTL on the mediator or the *cis*-eGene; "b" indicates the effects of the *cis*-eGene on the *trans*-eGene controlling for the effect of the corresponding eQTL; "Mediation Effect (%)" indicates the indirect effect of the eQTL on the *trans*-eGene, which is a×b, with the proportion of mediation effect among total effect in brackets. Benjamini-Hochberg FDR controlling procedure was applied to correct for multiple testing.

Supplementary Table 3: P-values of the enrichment analysis in Supplementary Figure 6.

	Resting M	LPS M	Resting T	PHA T
Allergic disease	6.3E-245	1.6E-156	8.3E-108	2.0E-74
Allergic rhinitis	0.999	0.998	0.997	0.996
Allergic sensitisation	0.974	0.971	0.970	0.570
Asthma	3.8E-10	1.7E-07	1.7E-15	5.7E-09
Asthma (adult)	1.1E-49	1.6E-46	1.4E-41	8.0E-29
Asthma (childhood)	1.1E-61	8.2E-51	3.3E-34	3.6E-28
IBD	5.1E-168	1.2E-102	2.3E-149	1.8E-150
Crohn's disease	4.7E-06	2.2E-26	6.6E-53	2.8E-82
Ulcerative colitis	1.8E-248	1.0E-195	2.8E-165	5.0E-199
Celiac disease	0.999	0.998	0.033	0.118
Celiac disease (ic)	4.3E-09	6.0E-06	2.9E-09	1.6E-07
ATD (ic)	1.5E-44	1.3E-37	8.8E-43	4.7E-112
JIA (ic)	6.9E-10	3.1E-06	1.2E-04	1.3E-03
Multiple sclerosis (ic)	1.9E-09	6.0E-09	4.0E-10	8.1E-06
Narcolepsy (ic)	3.9E-05	6.7E-04	0.014	0.998
PBC	4.1E-165	2.8E-152	7.4E-112	2.6E-126
PBC (ic)	1.4E-17	1.5E-09	5.3E-07	4.0E-13
PSC	9.9E-20	2.2E-19	6.3E-25	1.9E-24
Psoriasis (ic)	1.9E-04	8.3E-04	1.1E-05	5.8E-06
RA	1.2E-32	7.7E-35	4.4E-34	1.8E-31
RA (ic)	7.0E-13	2.1E-07	3.4E-06	2.5E-10
SLE	4.0E-56	8.4E-50	6.6E-47	2.4E-50
Type 1 diabetes	6.0E-192	2.2E-195	1.7E-182	3.7E-292
Type 1 diabetes (ic)	2.5E-21	3.5E-27	6.3E-12	9.5E-05
Edu attainment	1.2E-03	0.938	2.6E-04	6.9E-08

This table contains the exact P-values calculated from the enrichment analysis. Enrichment was tested for each of the four sets of *cis*-eQTLs separately ("M" and "T" indicate myeloid cells and T cells, respectively). The enrichment effect sizes and confidence intervals are in **Supplementary Figure 6**. The enrichment was estimated using significant eSNPs identified in resting myeloid cells (n=9,116 SNPs), LPS-stimulated myeloid cells (n=24,255 SNPs), resting T cells (n=65,147 SNPs), and PHA-stimulated T cells (n=92,378 SNPs). P-values that are not significant after Bonferroni correction are in grey. We used genome-wide association studies investigating allergic diseases (asthma, hay fever, or eczema)[1], allergic rhinitis[2], allergic sensitisation[2], asthma[3], adult-onset asthma[4], childhood-onset asthma[4], Inflammatory bowel disease (IBD)[5], Crohn's disease[5], and ulcerative colitis[5], celiac disease[6,7], autoimmune thyroid disease (ATD)[8], juvenile idiopathic arthritis (JIA)[9], multiple scleraosis[10], narcolepsy[11], primary biliary cirrhosis (PBC)[12,13], primary sclerosing cholangitis (PSC)[14], psoriasis[15], rheumatoid arthritis (RA)[16,17], systemic lupus erythematosus (SLE)[18], type 1 diabetes[19,20], and educational attainment[21] that serves as a negative control. Each row represents one disease, with "ic" indicating that the study was performed using ImmunoChip array.

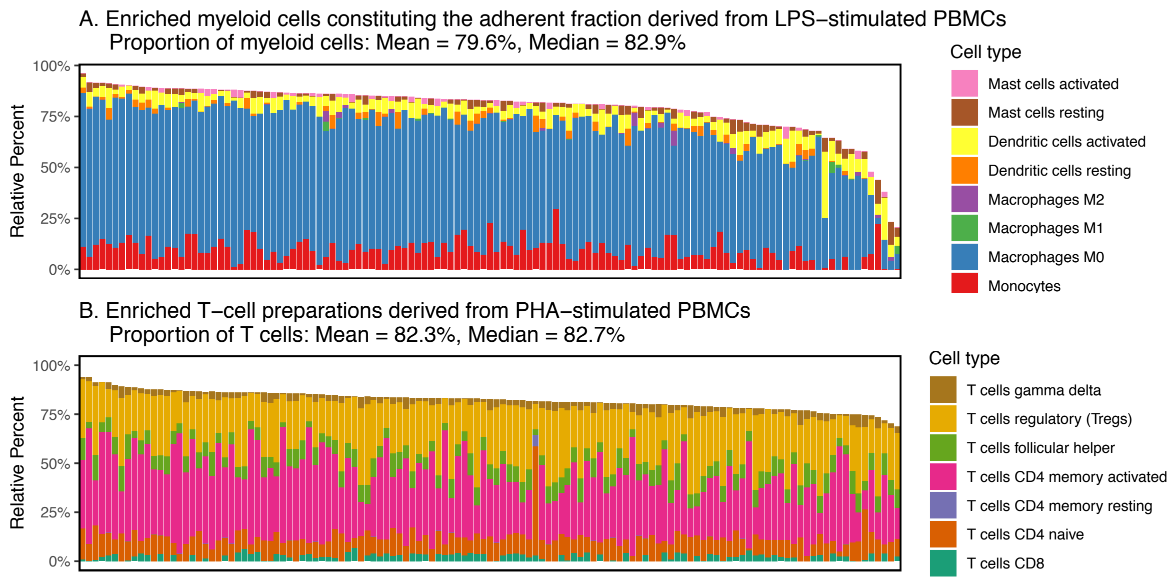

Supplementary Figure 1: Inferred cell type abundances from gene expression data. We performed tissue deconvolution analysis using CIBERSORTx (web-based) to estimate the cell type abundances of samples from four cell cultures. Relevant cell types are shown in the plots: panel **A** (LPS-stimulated myeloid-cell-enriched cultures) shows the estimated proportions of all myeloid cells that are available in the CIBERSORTx reference transcriptome profiles, namely monocytes, macrophages, dendritic cells, and mast cells; panel **B** shows the estimated proportions of various T cell types in the PHA-stimulated T-cell-enriched cultures. Results of resting cultures, which are not shown here, also were estimated to have high proportions: median=70.3% in resting myeloid-cell-enriched cultures and median=75.8% in resting T-cell-enriched cultures. Each bar in the plots represent one sample, and colours indicate different inferred cell types. Gene expression data that were quantile normalised across all 557 samples that passed QC from four cultures were used, and samples that were included in the eQTL analysis are shown here.

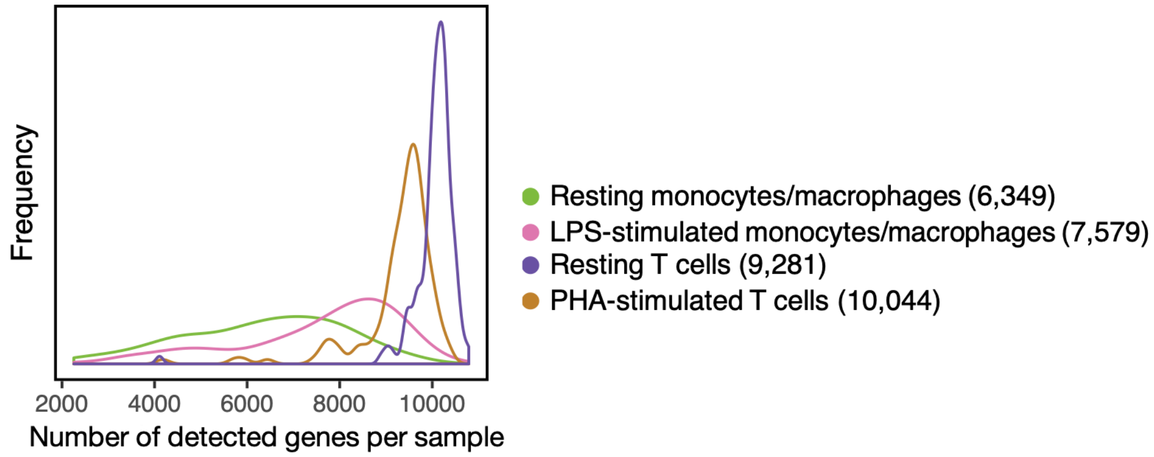

Supplementary Figure 2: Distribution of the number of detected genes per microarray sample. Colour indicates four experimental conditions. Number in brackets indicates the average number of detected genes (**Methods**) within each group.

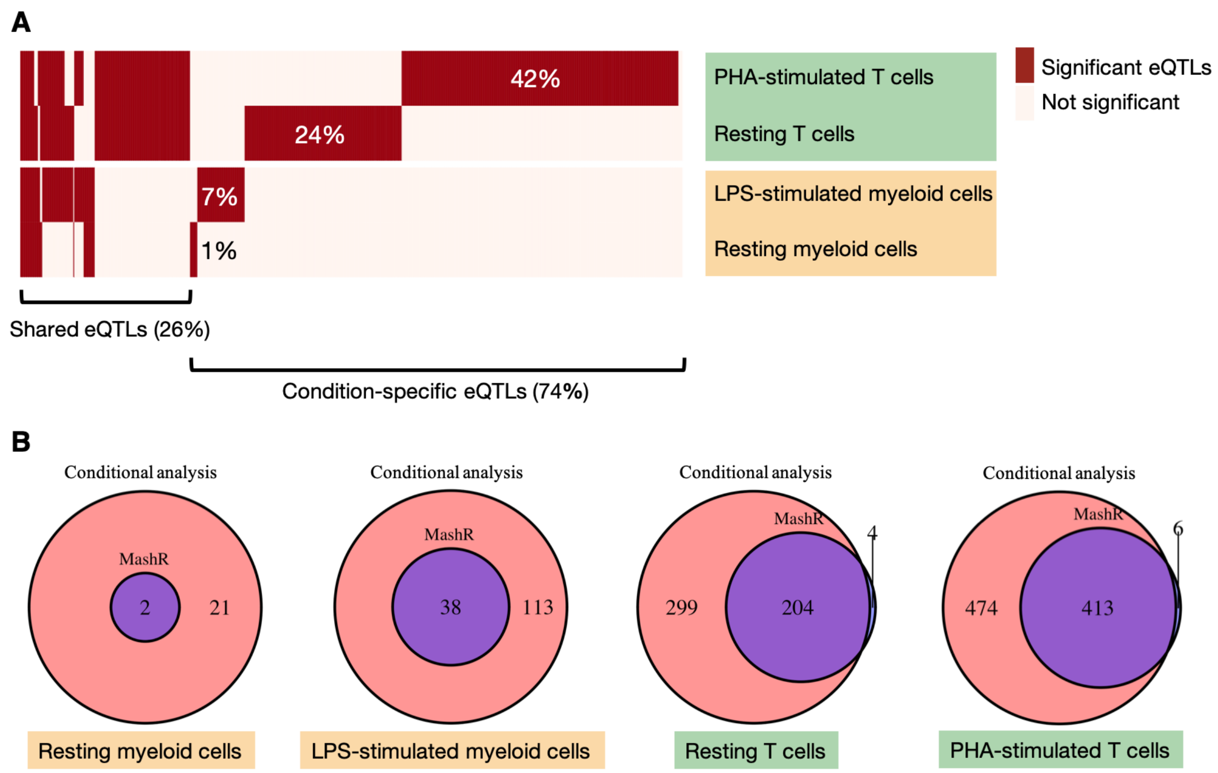

Supplementary Figure 3: Condition specificity of eQTLs. (**A**) the heatmap shows eQTL sharing across four experimental conditions (rows) using conditional analysis (**Methods**). Columns in the heatmap are unique eQTL–eGene associations. Significant associations are in red. Percentages labelled on the heatmap show the proportion of unique eQTL–eGene associations that are specific to a certain cell type and stimulatory condition. We also used a multivariate adaptive shrinkage (mash) model to compare eQTL signals across the four datasets (**Methods**), and the Venn diagrams (**B**) show the overlap of the condition-specific signals identified using these two methods.

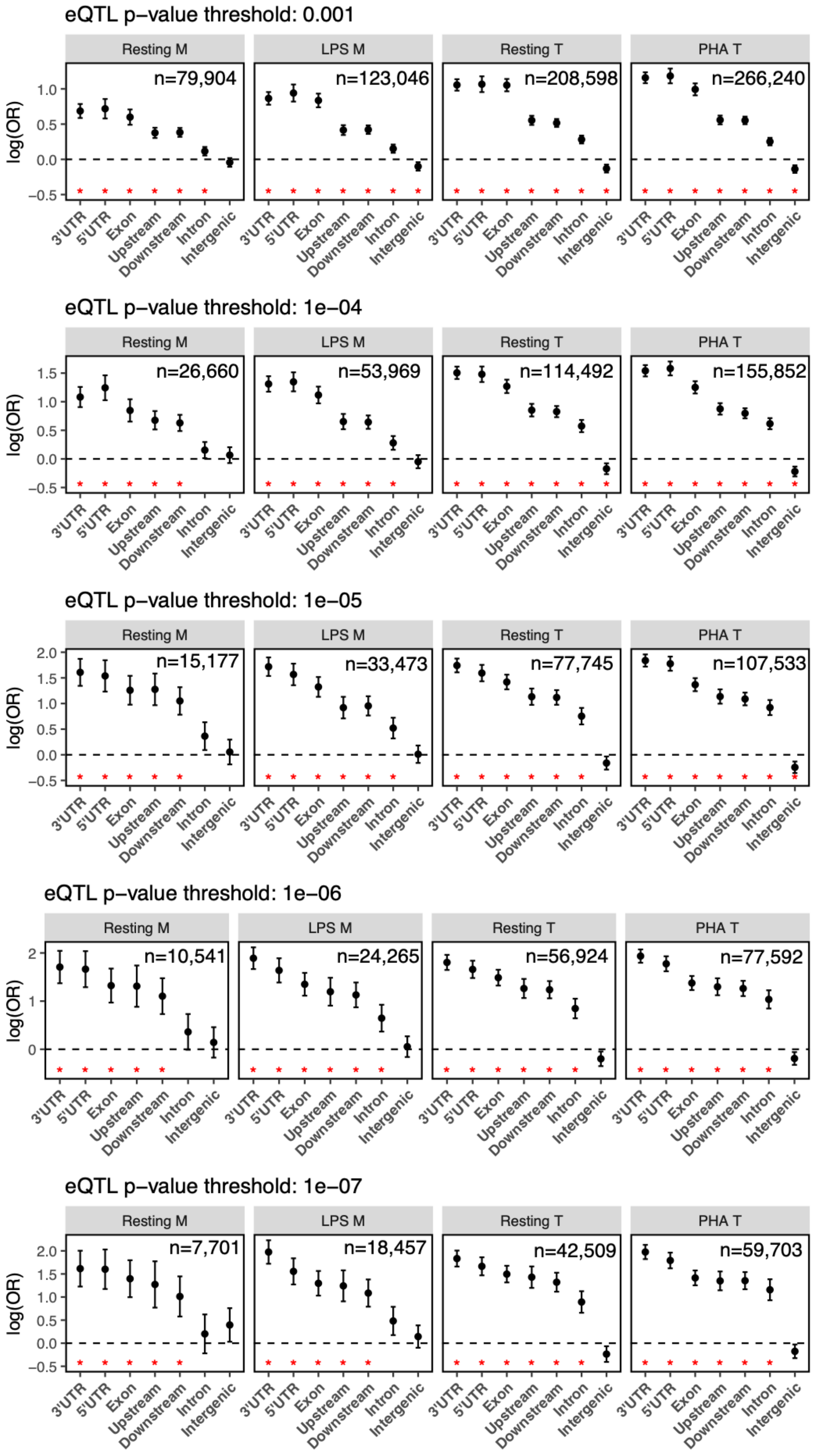
eQTL p−value threshold: 0.001
Resting M
n=79,904
LPS M
n=123,046
Resting T
n=208,598
PHA T
n=266,240
log(OR)
1.0
0.5
0.0
−0.5
3'UTR
5'UTR
Exon
Upstream
Downstream
Intron
Intergenic
eQTL p−value threshold: 1e−04
Resting M
n=26,660
LPS M
n=53,969
Resting T
n=114,492
PHA T
n=155,852
log(OR)
1.5
1.0
0.5
0.0
−0.5
3'UTR
5'UTR
Exon
Upstream
Downstream
Intron
Intergenic
eQTL p−value threshold: 1e−05
Resting M
n=15,177
LPS M
n=33,473
Resting T
n=77,745
PHA T
n=107,533
log(OR)
2.0
1.5
1.0
0.5
0.0
−0.5
3'UTR
5'UTR
Exon
Upstream
Downstream
Intron
Intergenic
eQTL p−value threshold: 1e−06
Resting M
n=10,541
LPS M
n=24,265
Resting T
n=56,924
PHA T
n=77,592
log(OR)
2
1
0
3'UTR
5'UTR
Exon
Upstream
Downstream
Intron
Intergenic
eQTL p−value threshold: 1e−07
Resting M
n=7,701
LPS M
n=18,457
Resting T
n=42,509
PHA T
n=59,703
log(OR)
2.0
1.5
1.0
0.5
0.0
−0.5
3'UTR
5'UTR
Exon
Upstream
Downstream
Intron
Intergenic

Supplementary Figure 4: Enrichment of *cis*-eQTLs in 3'UTR, 5'UTR, and exon regions. Data are presented as logarithm of Odds Ratio (OR), estimated at various eQTL significance levels from 1×10^{-3} to 1×10^{-7}, and its 95% confidence intervals (CIs). The number of SNPs that were used to estimate OR and CIs are shown in the plot. *GARFILED* accepts only one p-value threshold while the nominal p-value thresholds used to determine significant eQTLs vary across genes. In each row, four plots show the enrichment of *cis*-eQTL SNPs identified in four experimental conditions ("M" and "T" indicate myeloid cells and T cells, respectively). Tests that were significant after the Bonferroni correction are marked by red asterisks, where a P-value threshold of 0.0018 was used, adjusting for the 7×4 (28) tests.

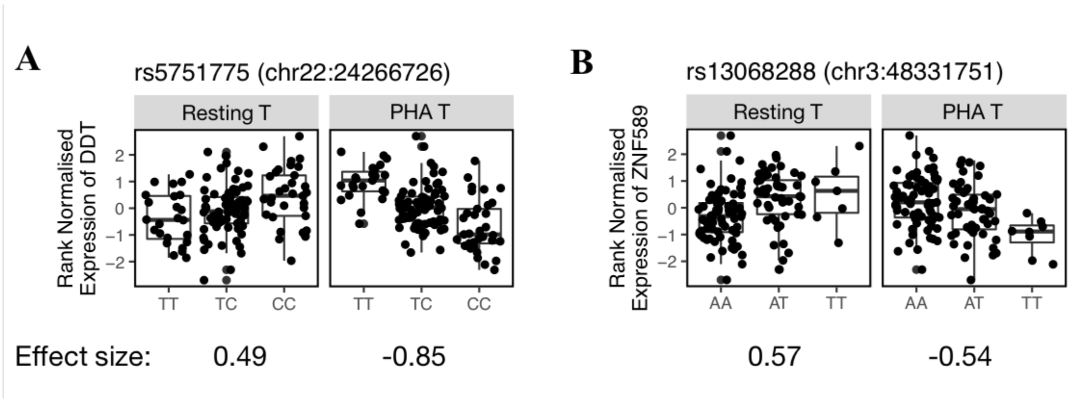

Supplementary Figure 5: Response eQTLs (reQTLs) with flipped directions of effects on gene expression across conditions. ReQTLs of *DDT* (**A**) and *ZNF585* (**B**) show opposite directions of eQTL effects. (**A**) Two boxplots show associations of rs5751775 (top eSNP in resting T cells) with *DDT* expression in resting (left) and PHA-stimulated (right) T cells. Dots indicate individuals stratified by genotypes (x-axes) and the rank-normalised gene expression is shown on y-axes. The centre line corresponds to median, and the lower and upper hinges indicate the 25th and 75th percentiles. The upper whisker extends from the hinge to the largest value ≤1.5 * inter-quartile range (IQR) from the hinge, and the lower whisker extends from the hinge to the smallest value at most 1.5 * IQR of the hinge. The highest and lowest dots show the maximum and minimum gene expression. Similarly, panel (**B**) shows the associations of rs13068288 (top eSNP in PHA-stimulated T cells) with *ZNF589* expression. Chromosome number and genomic position in hg19/GRCh37 are in brackets next to the SNP rsID. Effect sizes of these associations are shown under each boxplot, which is the number of s.d. of gene expression increased per allele dosage. For both panels, 126 and 127 biologically independent samples were used to identify eQTLs and estimate effect sizes in resting and PHA-stimulated T cells, respectively.

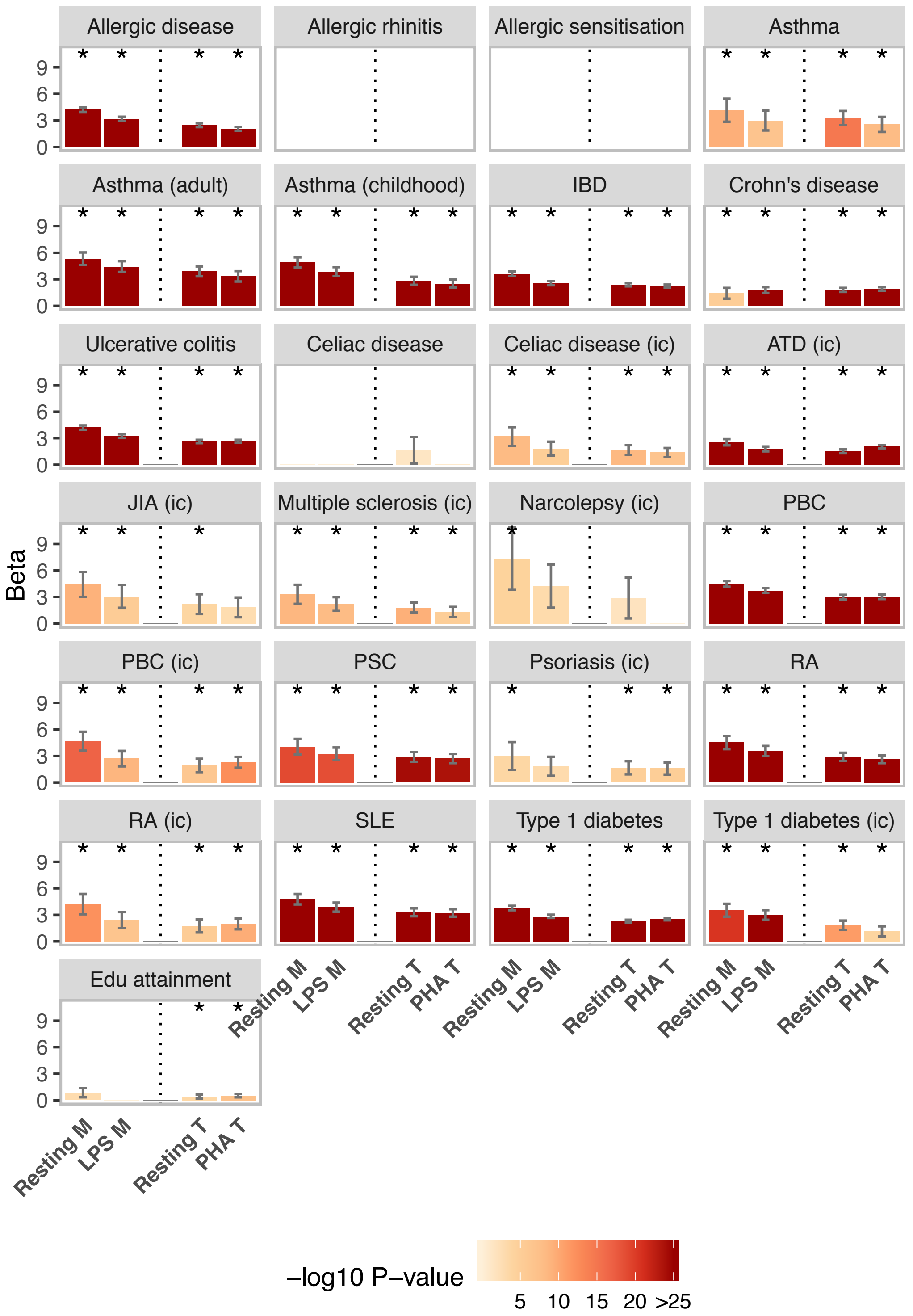

Supplementary Figure 6: Enrichment of early-life *cis*-eQTLs for genetic variants associated with immune-related diseases. We used genome-wide association studies investigating allergic diseases (asthma, hay fever, or eczema)[1], allergic rhinitis[2], allergic sensitisation[2], asthma[3], adult-onset asthma[4], childhood-onset asthma[4], Inflammatory bowel disease (IBD)[5], Crohn's disease[5], and ulcerative colitis[5], celiac disease[6,7], autoimmune thyroid disease (ATD)[8], juvenile idiopathic arthritis (JIA)[9], multiple scleraosis[10], narcolepsy[11], primary biliary cirrhosis (PBC)[12,13], primary sclerosing cholangitis (PSC)[14], psoriasis[15], rheumatoid arthritis (RA)[16,17], systemic lupus erythematosus (SLE)[18], type 1 diabetes[19,20], and educational attainment[21] that serves as a negative control. Each plot represents one disease, with "ic" indicating that the study was performed using ImmunoChip array. Enrichment was tested for each of the four sets of *cis*-eQTLs separately

(x-axes: "M" and "T" indicate myeloid cells and T cells, respectively). Height of each bar indicates the enrichment effect size or logarithm of the odds ratio (centre of error bars) with 95% confidence intervals (CIs) shown as the error bars. The enrichment was estimated using significant eSNPs identified in resting myeloid cells (n=9,116 SNPs), LPS-stimulated myeloid cells (n=24,255 SNPs), resting T cells (n=65,147 SNPs), and PHA-stimulated T cells (n=92,378 SNPs). Tests that are not significant at P-value 0.05 are not shown since the beta estimates are not reliable and the CIs are too large. Colour of each bar indicates the significance level based on minus log10 P-values; the same colour is used if the P-value is smaller than 1×10^{-25} (the exact P-values are in **Supplementary Table 3**). Asterisks indicate that the tests are significant after Bonferroni correction for multiple testing.

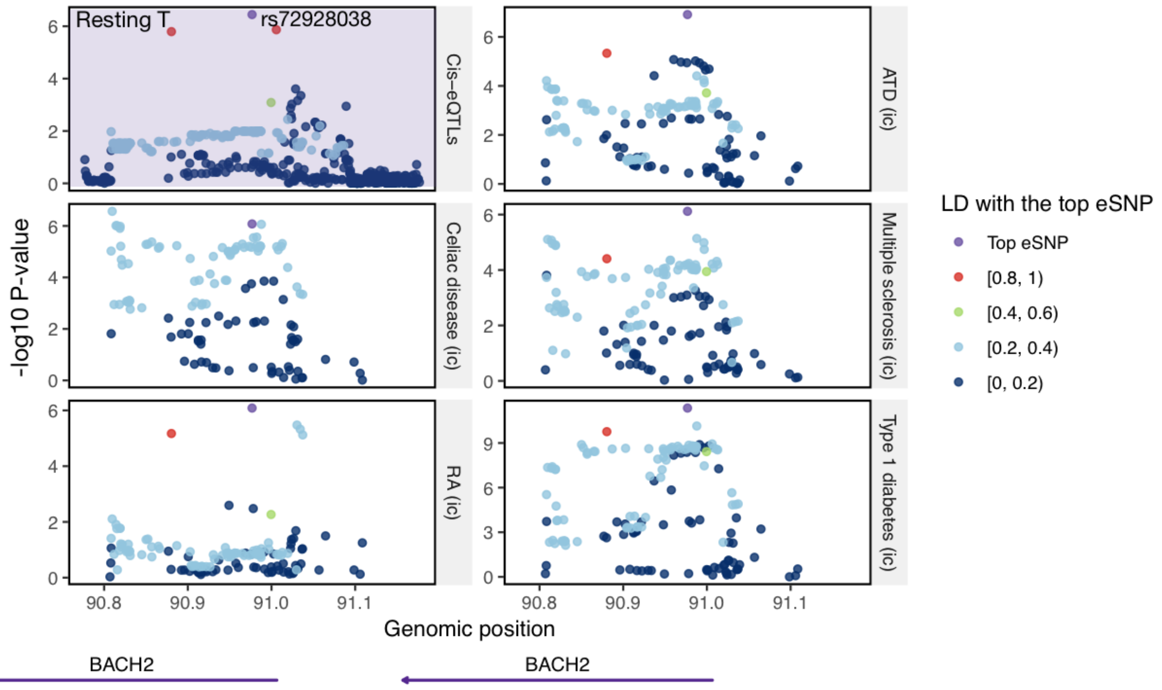

Supplementary Figure 7: Colocalisation of the *cis*-eQTL of *BACH2* with multiple diseases. Six regional plots show eQTL association with *BACH2* expression in resting T cells (purple background), and GWAS associations with autoimmune thyroid disease (ATD), celiac disease, multiple sclerosis, rheumatoid arthritis (RA), and type 1 diabetes (white background). "ic" indicates that the GWAS was performed using ImmunoChip array. The minus log10 P-value is plotted on y-axes for SNPs located within 200 kb from the top eSNP of *BACH2* (rs72928038). The regional plots for GWAS signals show SNPs that are available in both datasets (i.e. SNPs that were tested in the colocalisation analysis), and the first eQTL plot shows all SNPs in the CAS dataset. Colours of dots indicate the LD r^2 correlation with the top eSNP (purple). Positions of genes located on this locus are shown at the bottom. *BACH2* did not have significant *cis*-eQTLs in the other three experimental conditions. We used n=126 biologically independent samples to identify eQTLs and estimate effect sizes in resting T cells.

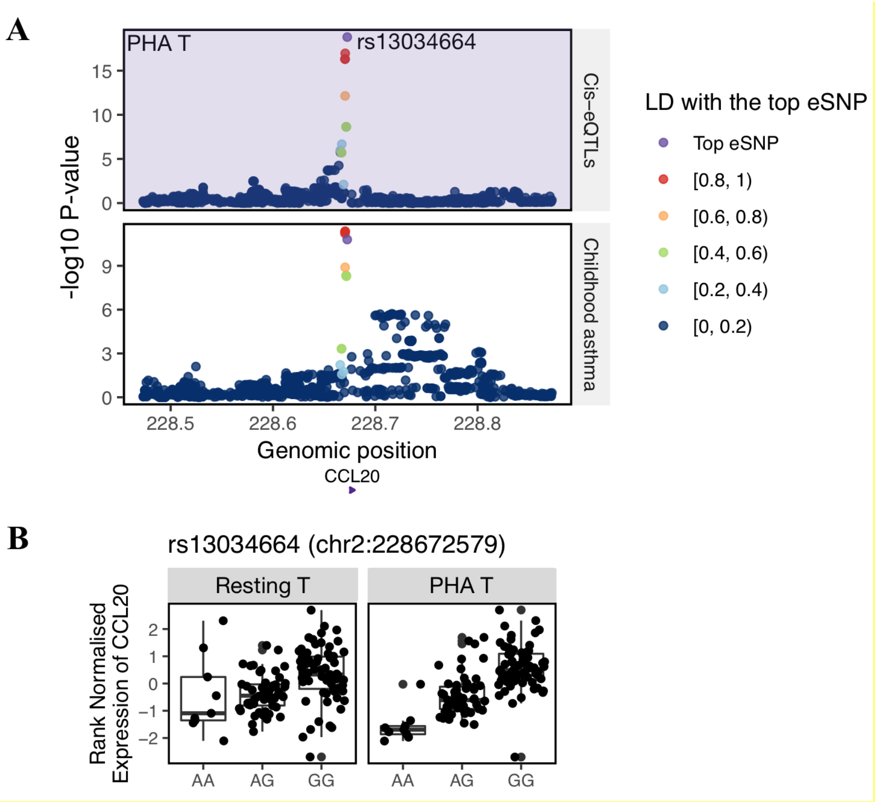

Supplementary Figure 8: Colocalisation between the response eQTL (reQTL) of *CCL20* and childhood-onset asthma association. (**A**) Regional plots show eQTL association with *CCL20* expression in PHA-stimulated T cells (purple background), and GWAS association with childhood-onset asthma (white background). The minus log10 P-value is plotted on y-axes for SNPs located within 200 kb from the top eSNP of *CCL20* (rs13034664). The second plot for the GWAS signal shows SNPs that are available in both datasets (i.e. SNPs that were tested in the colocalisation analysis), and the first eQTL plot shows all SNPs in the CAS dataset. Colours of dots indicate the LD r^2 correlation with the top eSNP (purple) based on CAS genotype data. Positions of genes located on this locus are shown at the bottom. *CCL20* did not have significant *cis*-eQTLs in the other three experimental conditions. (**B**) Boxplots show the rank-normalised *CCL20* expression (y-axes) in resting (left) and stimulated (right) T cells stratified by genotypes of the reQTL (x-axes). The centre line corresponds to median, and the lower and upper hinges indicate the 25th and 75th percentiles. The upper whisker extends from the hinge to the largest value ≤1.5 * inter-quartile range (IQR) from the hinge, and the lower whisker extends from the hinge to the smallest value at most 1.5 * IQR of the hinge. The highest and lowest dots show the maximum and minimum gene expression. In resting T cells, no SNP was significantly associated with *CCL20*. We used n=126 and 127 biologically independent samples to identify eQTLs and estimate effect sizes in resting and PHA-stimulated T cells, respectively.

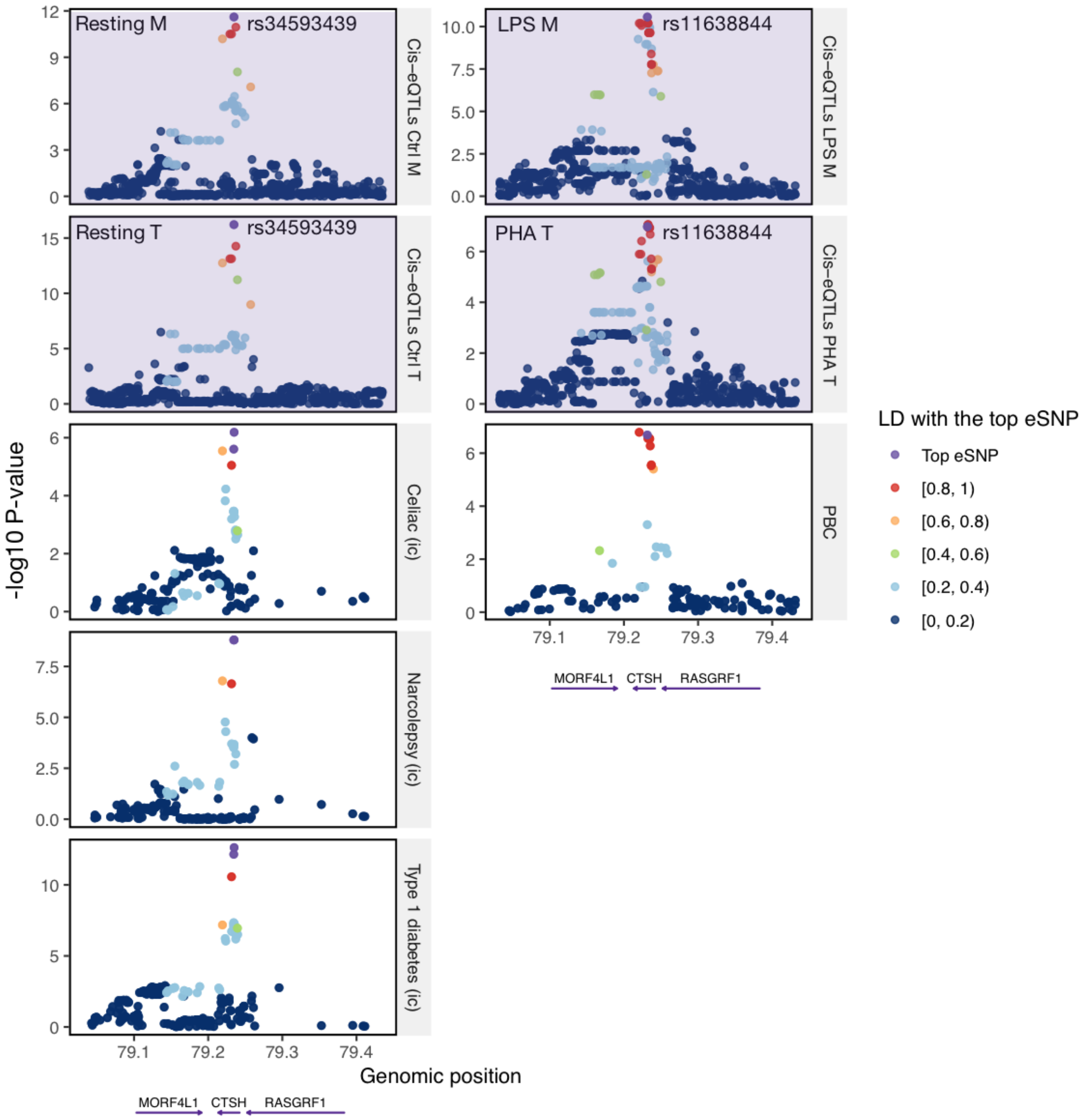

Supplementary Figure 9: Colocalisations with different diseases are observed for the *CTSH cis*-eQTLs identified in resting (left) and stimulated (right) cells. On the left, regional plots show eQTL associations (purple background) with *CTSH* expression identified in resting myeloid cells (Resting M) and resting T cells (Resting T). The two eQTL signals have the same top eSNP rs34593439, and both colocalised with GWAS hits associated with celiac disease, narcolepsy, and type 1 diabetes (white background). "ic" indicates that the GWAS was performed using ImmunoChip array. On the right, regional plots show eQTL associations (purple background) with *CTSH* expression identified in LPS-stimulated myeloid cells (LPS M; top eSNP rs11638844) and PHA-stimulated T cells (PHA T). They both colocalised with GWAS signal for primary biliary cirrhosis (PBC; white background). The minus log10 P-value is plotted on y-axes for SNPs located within 200 kb from the corresponding top eSNP (left: rs34593439; right: rs11638844). The regional plots for GWAS signals show SNPs that are available in both datasets (i.e. SNPs that were tested in the colocalisation analysis), and the regional plots for eQTLs show all SNPs in the CAS dataset. Colours of dots indicate the LD r^2 correlation with the top eSNP (purple). Positions of genes located on this locus are shown at the bottom. We used n=116, 125, 126 and 127 biologically independent samples to identify eQTLs and estimate effect sizes in resting myeloid cells, LPS-stimulated myeloid cells, resting T cells, and PHA-stimulated T cells, respectively.

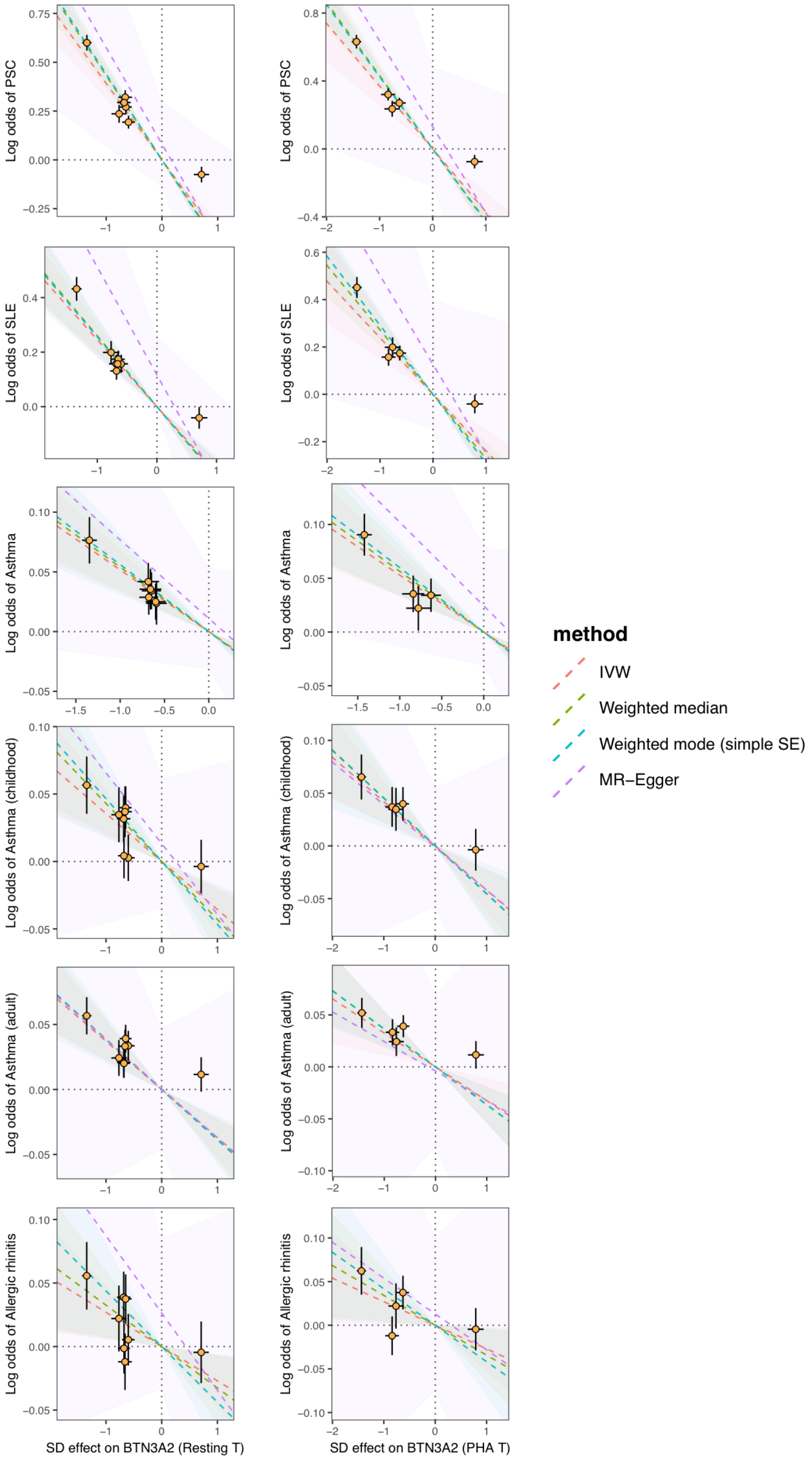

Supplementary Figure 10: Causal associations (negative) between *BTN3A2* expression and immune-related diseases. Data are presented as *cis*-eQTL effect sizes (centre) with 95% confidence intervals (CIs) on x-axes and genetic effect sizes of SNPs (centre) on disease risk with 95% CIs on y-axes. *Cis*-eQTL effect sizes were estimated from resting T cells (left; n=126 biologically independent samples) and PHA-stimulated T cells (right; n=127). Rows show immune-related diseases that were negatively associated with *BTN3A2* expression (PSC: primary sclerosing cholangitis; SLE: systemic lupus erythematosus). In each plot, four

fitted lines indicate different Mendelian randomisation methods and ribbons indicate their 95% confidence intervals (IVW: inverse variance weighted; **Methods**).

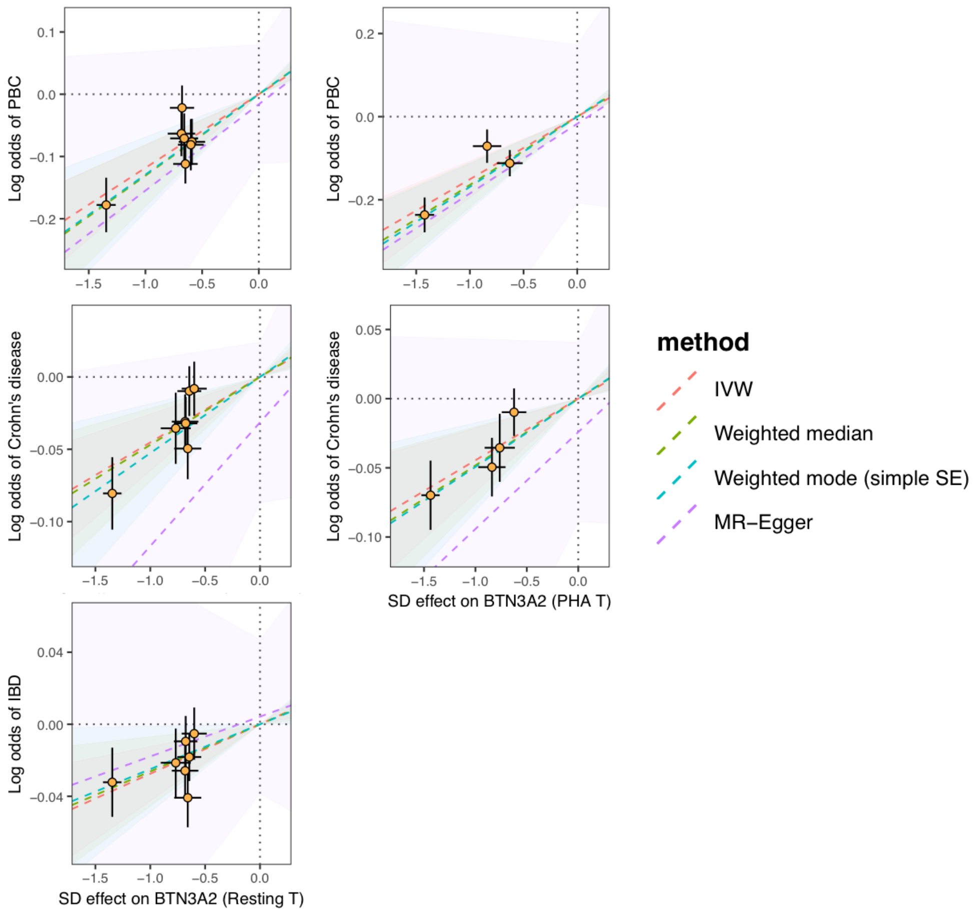

Supplementary Figure 11: Causal associations (positive) between *BTN3A2* expression and immune-related diseases. Data are presented as *cis*-eQTL effect sizes (centre) with 95% confidence intervals (CIs) on x-axes and genetic effect sizes of SNPs (centre) on disease risk with 95% CIs on y-axes. *Cis*-eQTL effect sizes were estimated from resting T cells (left; n=126 biologically independent samples) and PHA-stimulated T cells (right; n=127). Rows show immune-related diseases that were positively associated with *BTN3A2* expression (PBC: primary biliary cirrhosis; IBD: inflammatory bowel disease). In each plot, four fitted lines indicate different Mendelian randomisation methods and ribbons indicate their 95% confidence intervals (IVW: inverse variance weighted; **Methods**). The plot for causal effects of *BTN3A2* expression in stimulated T cells on IBD is missing because the number of available genetic instruments was too low (<3) to perform various Mendelian randomisation methods.

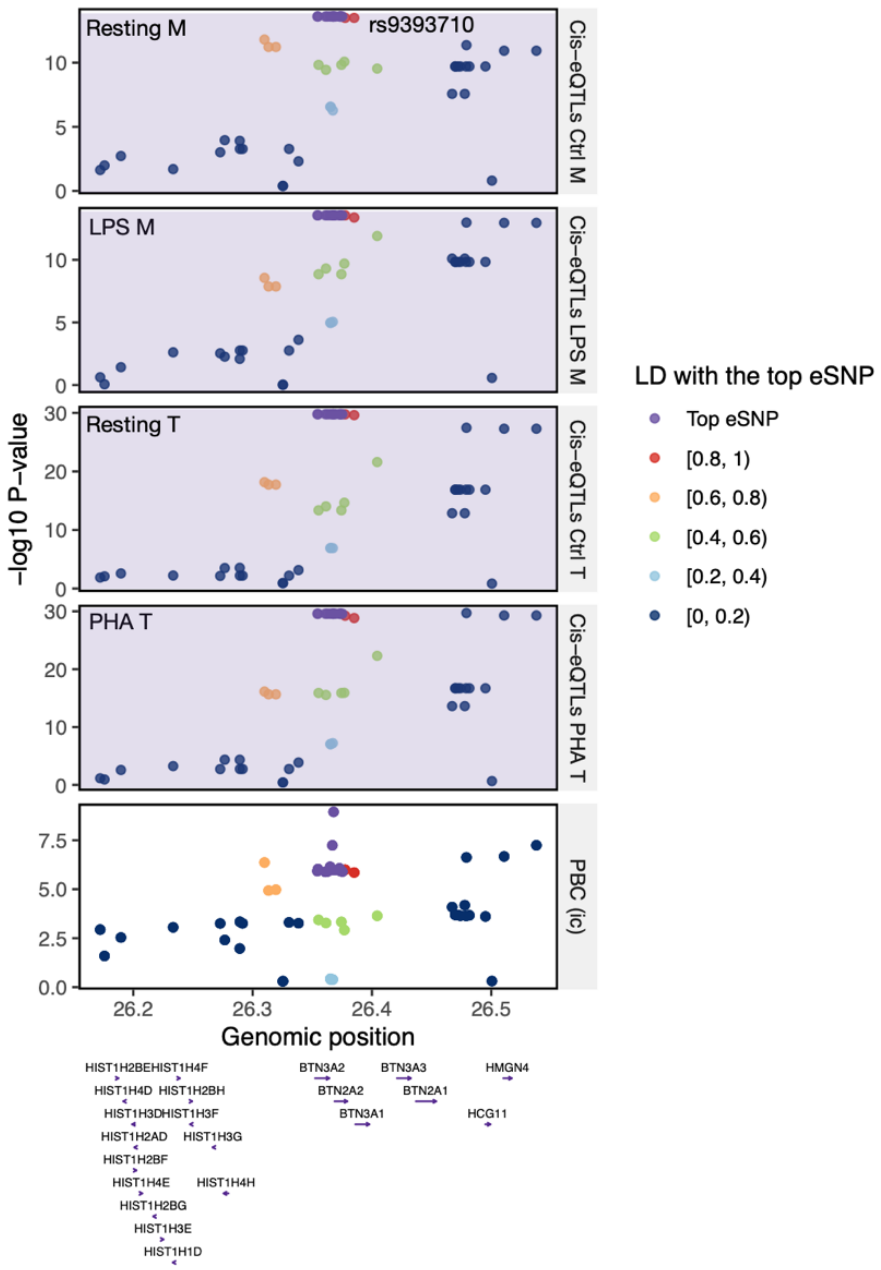

Supplementary Figure 12: Colocalisation between the *cis*-eQTLs of *BTN3A2* in four experimental conditions with primary biliary cirrhosis (PBC). Five regional plots show eQTL associations (purple background) with *BTN3A2* expression in four conditions ("M" and "T" indicate myeloid cells and T cells, respectively), and GWAS association with PBC (white background). "ic" indicates that the GWAS was performed using ImmunoChip array. The minus log10 P-value is plotted on y-axes for SNPs located within 200 kb from rs9393710, which was the top SNP of the GWAS hit. rs9393710 was among the top eSNPs identified in resting and stimulated myeloid cells and in resting T cells, and the top eSNP in stimulated T cells was in high LD with it ($r^2 = 0.92$). The regional plot for the GWAS signal shows SNPs that are available in both datasets (i.e. SNPs that were tested in the colocalisation analysis), and the regional plots for eQTLs show all SNPs in the CAS dataset. Colours of dots indicate the LD r^2 correlation with the top eSNP (purple) based on CAS genotype data. Positions of genes located on this locus are shown at the bottom. We used n=116, 125, 126 and 127 biologically independent samples to identify eQTLs and estimate effect sizes in resting myeloid cells, LPS-stimulated myeloid cells, resting T cells, and PHA-stimulated T cells, respectively.

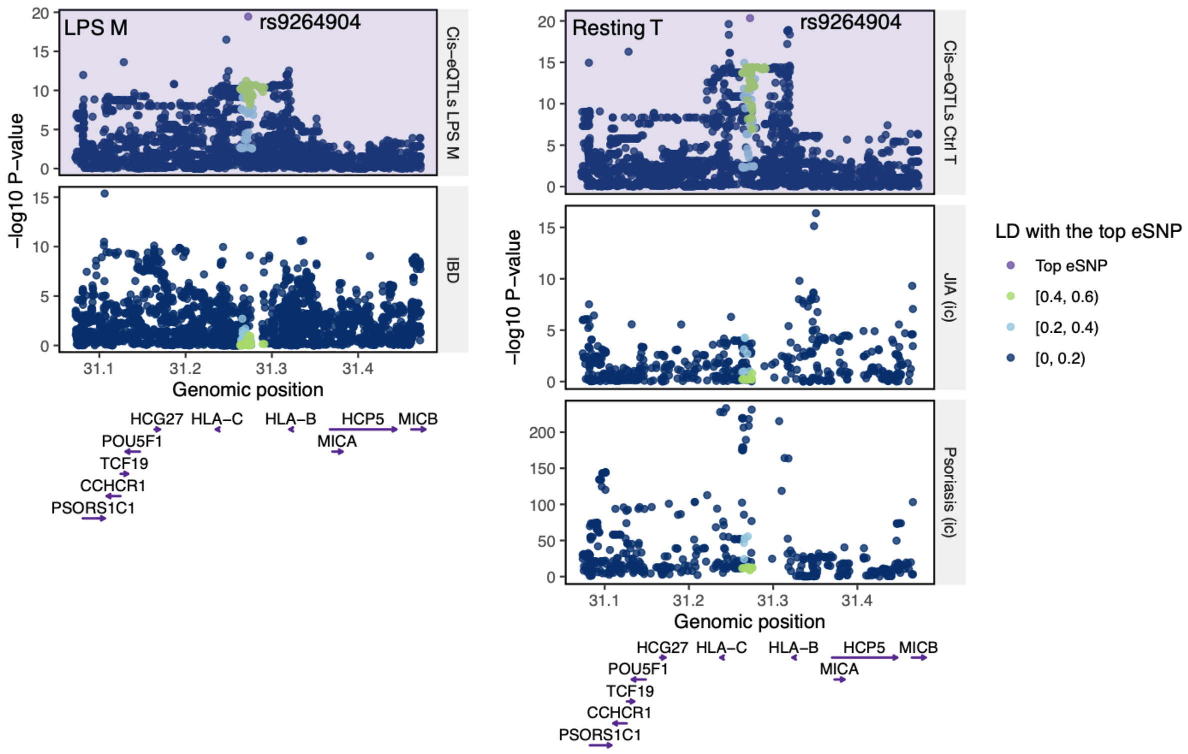

Supplementary Figure 13: Regional plot of the *cis*-eQTL of *HLA-C* and GWAS signals on the same locus. We plotted the diseases that were significantly associated with *HLA-C* expression in Mendelian Randomisation analysis. The top two plots show the eQTL associations (purple background) in LPS-stimulated myeloid cells, and resting T cells, which share the same lead eQTL SNP (rs9264904). The rest three plots show GWAS associations with inflammatory bowel disease (IBD), juvenile idiopathic arthritis (JIA) and Psoriasis (white background), and "ic" indicates that the GWAS was performed using ImmunoChip array. The minus log10 P-value is plotted on y-axes for SNPs located within 200 kb from rs9264904. The regional plots for GWAS signals show SNPs that are available in both datasets (i.e. SNPs that were tested in the colocalisation analysis), and the regional plots for eQTLs show all SNPs in the CAS dataset. Colours of dots indicate the LD r^2 correlation with the top eSNP (purple) based on CAS genotype data. Positions of genes located on this locus are shown at the bottom. We used n=125 and 126 biologically independent samples to identify eQTLs and estimate effect sizes in LPS-stimulated myeloid cells and resting T cells, respectively.

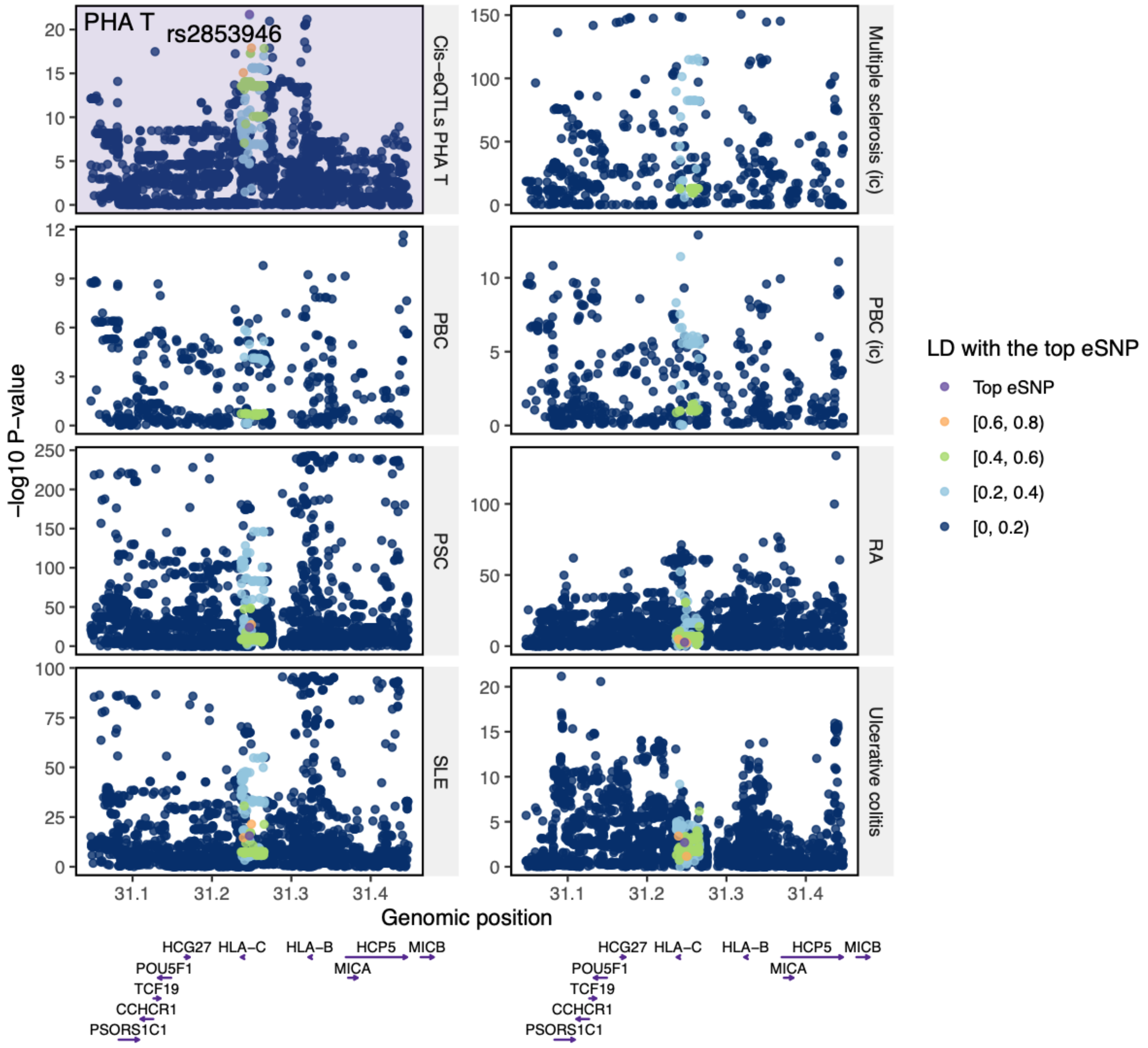

Supplementary Figure 14: Regional plot of the *cis*-eQTL of *HLA-C* and GWAS signals on the same locus. We plotted the diseases that were significantly associated with *HLA-C* expression in Mendelian Randomisation analysis. The first plot shows the eQTL association (purple background) in PHA-stimulated T cell, with the lead eQTL SNP (rs2853946) in purple. The rest regional plots show GWAS associations with multiple sclerosis, primary biliary cirrhosis (PBC), primary sclerosing cholangitis (PSC), rheumatoid arthritis (RA), systemic lupus erythematosus (SLE), and ulcerative colitis (white background), and "ic" indicates that the GWAS was performed using ImmunoChip array. The minus log10 P-value is plotted on y-axes for SNPs located within 200 kb from rs2853946. The regional plots for GWAS signals show SNPs that are available in both datasets (i.e. SNPs that were tested in the colocalisation analysis), and the regional eQTL plot shows all SNPs in the CAS dataset. Colours of dots indicate the LD r^2 correlation with the top eSNP (purple) based on CAS genotype data. Positions of genes located on this locus are shown at the bottom.

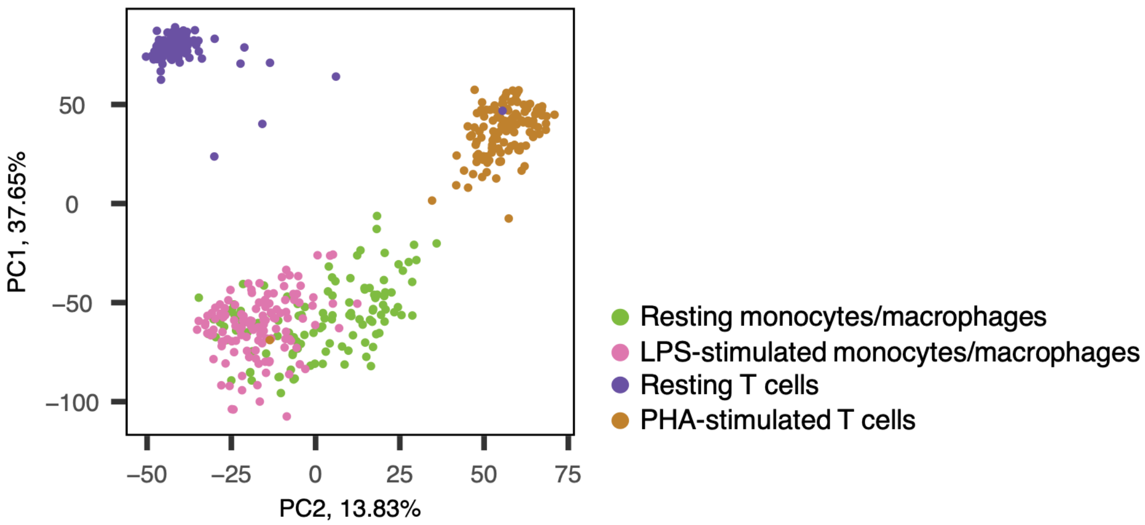

Supplementary Figure 15: Principal component analysis (PCA) of gene expression data. Each dot represents one sample from four different experimental conditions. Gene expression data used for PCA were quantile normalised across all 557 samples that passed QC. PCs were calculated using the 494 samples that are included the eQTL analysis (i.e. samples from individuals with genotype data available). First two PCs are plotted, and the proportion of variation explained by each PC is shown.

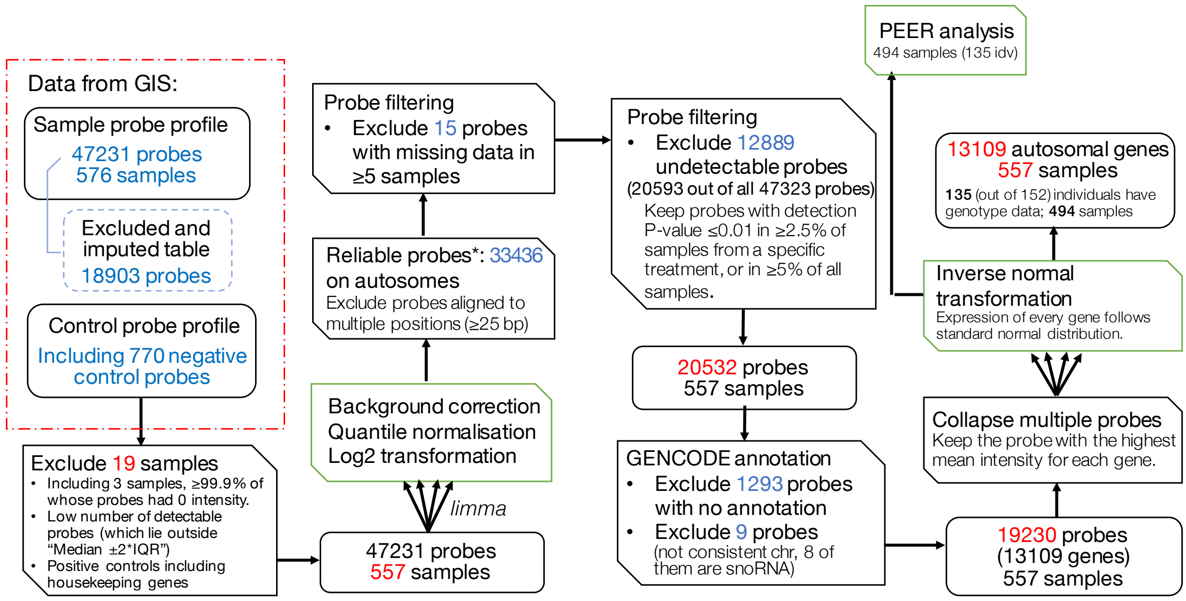

Supplementary Figure 16: QC and normalisation pipeline. Steps where data processing was performed for four cell cultures separately are in green box. There were 576 samples from 152 individuals that went through the Illumina microarray platform, and 557 of them passed QC steps. For eQTL analysis, we used 494 microarray samples from 135 individuals with genotype data available.

References:

1. Ferreira, M.A. *et al*. Shared genetic origin of asthma, hay fever and eczema elucidates allergic disease biology. *Nat Genet* **49**, 1752-1757 (2017).
2. Waage, J. *et al*. Genome-wide association and HLA fine-mapping studies identify risk loci and genetic pathways underlying allergic rhinitis. *Nat Genet* **50**, 1072-1080 (2018).
3. Demenais, F. *et al*. Multiancestry association study identifies new asthma risk loci that colocalize with immune-cell enhancer marks. *Nat Genet* **50**, 42-53 (2018).
4. Pividori, M., Schoettler, N., Nicolae, D.L., Ober, C. & Im, H.K. Shared and distinct genetic risk factors for childhood-onset and adult-onset asthma: genome-wide and transcriptome-wide studies. *Lancet Respir Med* **7**, 509-522 (2019).
5. de Lange, K.M. *et al*. Genome-wide association study implicates immune activation of multiple integrin genes in inflammatory bowel disease. *Nat Genet* **49**, 256-261 (2017).
6. Dubois, P.C. *et al*. Multiple common variants for celiac disease influencing immune gene expression. *Nat Genet* **42**, 295-302 (2010).
7. Trynka, G. *et al*. Dense genotyping identifies and localizes multiple common and rare variant association signals in celiac disease. *Nat Genet* **43**, 1193-201 (2011).
8. Cooper, J.D. *et al*. Seven newly identified loci for autoimmune thyroid disease. *Hum Mol Genet* **21**, 5202-8 (2012).
9. Hinks, A. *et al*. Dense genotyping of immune-related disease regions identifies 14 new susceptibility loci for juvenile idiopathic arthritis. *Nat Genet* **45**, 664-9 (2013).
10. International Multiple Sclerosis Genetics, C. *et al*. Analysis of immune-related loci identifies 48 new susceptibility variants for multiple sclerosis. *Nat Genet* **45**, 1353-60 (2013).
11. Faraco, J. *et al*. ImmunoChip study implicates antigen presentation to T cells in narcolepsy. *PLoS Genet* **9**, e1003270 (2013).
12. Cordell, H.J. *et al*. International genome-wide meta-analysis identifies new primary biliary cirrhosis risk loci and targetable pathogenic pathways. *Nat Commun* **6**, 8019 (2015).
13. Liu, J.Z. *et al*. Dense fine-mapping study identifies new susceptibility loci for primary biliary cirrhosis. *Nat Genet* **44**, 1137-41 (2012).
14. Ji, S.G. *et al*. Genome-wide association study of primary sclerosing cholangitis identifies new risk loci and quantifies the genetic relationship with inflammatory bowel disease. *Nat Genet* **49**, 269-273 (2017).
15. Tsoi, L.C. *et al*. Identification of 15 new psoriasis susceptibility loci highlights the role of innate immunity. *Nat Genet* **44**, 1341-8 (2012).
16. Okada, Y. *et al*. Genetics of rheumatoid arthritis contributes to biology and drug discovery. *Nature* **506**, 376-81 (2014).
17. Eyre, S. *et al*. High-density genetic mapping identifies new susceptibility loci for rheumatoid arthritis. *Nat Genet* **44**, 1336-40 (2012).
18. Bentham, J. *et al*. Genetic association analyses implicate aberrant regulation of innate and adaptive immunity genes in the pathogenesis of systemic lupus erythematosus. *Nat Genet* **47**, 1457-1464 (2015).
19. Bradfield, J.P. *et al*. A genome-wide meta-analysis of six type 1 diabetes cohorts identifies multiple associated loci. *PLoS Genet* **7**, e1002293 (2011).
20. Onengut-Gumuscu, S. *et al*. Fine mapping of type 1 diabetes susceptibility loci and evidence for colocalization of causal variants with lymphoid gene enhancers. *Nat Genet* **47**, 381-6 (2015).
21. Lee, J.J. *et al*. Gene discovery and polygenic prediction from a genome-wide association study of educational attainment in 1.1 million individuals. *Nat Genet* **50**, 1112-1121 (2018).
