# Supplementary material for: Neonatal genetics of gene expression reveal potential origins of autoimmune and allergic disease risk
Source: Nat Commun. 2020 Jul 28;11:3761. doi: 10.1038/s41467-020-17477-x (PMC7387553; doi:10.1038/s41467-020-17477-x)
Supplement: Supplementary file 3 — Description of Supplementary Data files [file 41467_2020_17477_MOESM3_ESM.docx]

**Description of Additional Supplementary Data**

**Supplementary Data 1**

This file contains genes with *cis-*eQTLs (eGenes) and their lead SNPs and those in high LD identified in resting myeloid cells (sheet A), LPS-stimulated myeloid cells (sheet B), resting CD4^+^ T cells (sheet C), PHA-stimulated CD4^+^ T cells (sheet D). The "ReQTL" column indicates whether the eGene (the "Gene" column) had significant response eQTL; "P-value" and "Beta" were obtained from linear regression models. The "SNP" and "RSID" columns indicate the top eSNP for each eGene, and the GRCh37/hg19 genomic location is shown in the "Chr" and "Position" columns. The "Counted_allele" column indicates the allele based on which the effect size was calculated; "Other_allele" indicates the other allele; "Freq_counted" shows the frequency of the counted allele; "SNPs_perfectLD" shows all eSNPs that were in perfect LD with the top eSNP in CAS; "SNPs_highLD" shows the eSNPs that were in high LD (r^2^ ≥0.8) with the top eSNP.

**Supplementary Data 2**

This file contains response eQTLs (reQTLs) identified in myeloid cells (sheet A) and CD4^+^ T cells (sheet B). Interaction tests on top eSNPs (the "SNP", "RSID", "Chr", and "Position" columns) of significant eGenes (the "Gene" column) were performed to identify reQTLs. For eGenes that had significant *cis*-eQTLs in both resting and stimulated conditions (the "Condition" column), one top eSNP was tested if the two top eSNPs were in high LD (r^2^ ≥0.8) and both top eSNPs were tested if they were independent (LD r^2^ <0.8). "LD_othertop" shows the LD correlation between two top eSNPs; LD r^2^ was not calculated (NA) if the eGene had significant *cis*-eQTL in only one condition. The "Tested" column indicates three categories: "Single" – the gene had significant *cis*-eQTL in one of the two conditions; "Independent" – two top eSNPs were not in high LD; "High LD" – two top eSNPs were in high LD and one was kept. The "Condition" column indicates the condition where the reQTL was identified ("Stimulated" or "Resting"); if both conditions are listed, it means that the two top eSNPs were in high LD (same eQTL signal with different effect sizes) and the one in the first condition was tested. The "Beta_resting_lmer" and "Beta_interaction_lmer" columns show the effect size in resting condition and the effect size increased by stimulation, which were obtained from lmer linear mixed models; "PermPval" indicates the permutation adjusted P-values for the interaction term in lmer models (note that the minimum permutation P-value is 1/1001, since 1000 permutations were performed); "Counted_allele" indicates the allele based on which the effect size was calculated; "Other_allele" indicates the other allele; "Freq_counted" shows the frequency of the counted allele.

**Supplementary Data 3**

This file contains *trans*-eQTLs identified in resting and stimulated myeloid cells and CD4^+^ T cells. "SNP_chr" and "SNP_pos" show the genomic location (GRCh37/hg19) of the top eSNP ("RSID") of the *trans*-eGene; "Chr" and "TSS" show the chromosome number and the transcription start site of the *trans-*eGene; "P-value" and "Beta" were obtained from linear regression models; "R2_topeSNP" indicates the LD correlation between the eSNP and the top eSNP of the corresponding *trans*-eGene; "Counted_allele" indicates the allele based on which the beta was calculated; "Other_allele" indicates the other allele; "Freq_counted" shows the frequency of the counted allele; "*Cis*-eGene" shows all *cis*-eGenes associated with the *trans*-eSNP and an empty entry means that the corresponding eSNP was not significantly associated with any *cis*-eGenes.

**Supplementary Data 4**

This file contains significant colocalisations of neonatal *cis*-eQTLs with GWAS hits of immune-mediated diseases (“ic” indicates that the GWAS was performed using ImmunoChip array). Colocalised response eQTLs (“ReQTL”) are highlighted in orange. The “*Cis*-eGene” column shows the genes that are associated with the corresponding colocalised *cis*-eQTLs identified in resting or stimulated myeloid cells (“M”) and T cells (“T”), which were stimulated by either lipopolysaccharide (LPS) or phytohemagglutinin (PHA), respectively. The “Top_eSNP” (chromosome number followed by GRCh37/hg19 genomic position) and “RSID” columns show the lead *cis*-eQTL SNPs for each gene. The “TopeSNP_tested_in_coloc” column indicates whether the lead eSNP was also available in the corresponding GWAS dataset. The “Min_GWAS_Pval” column shows the minimum P-value of the corresponding locus. The number of SNPs in eQTL and GWAS datasets (“#SNPs_eQTL” and “#SNPs_GWAS”), and the number of overlapping SNPs (“#SNP_tested”) for each locus are shown. The “PP3_distinct" and “PP_shared” columns show the posterior probabilities for distinct and shared causal variants, respectively. The sum (PP_shared + PP_distinct) and the ratio (PP_shared/PP_distinct) are shown in the “Sum_PP” and “Ratio_PP” columns. The four columns (“Resting M”, “LPS M”, “Resting T”, “PHA T”) show the PP_shared between the GWAS signal and eQTLs in all four conditions. Colocalisation was not tested (indicated by “Not tested”) for conditions in which the gene was not a significant *cis-*eGene. PP_shared for conditions that were not considered as significant colocalisation is highlighted. The LD correlation (r^2^ and D') between the lead eQTL SNP (“Top_eSNP”) and the lead GWAS SNP (“Lead_GWAS_SNP”) for each locus is reported. The last five columns show if the colocalisation analysis passed the sensitivity analysis, and the PP_shared when using different priors in the colocalisation analysis: (1) default priors (p1=1e-4, p2=1e-4, p12=1e-5), (2) changing p1 from 1e-4 to 1e-5, (3) changing p2 from 1e-4 to 1e-5, and (4) changing both p1 and p2 to 1e-5. Note that we used p1=1e-4, p2=1e-4, p12=1e-6.

**Supplementary Data 5**

This file contains neonatal gene expression (“Exposure”) that were causally associated with multiple immune-mediated diseases using Mendelian Randomisation analysis (MR). References for the GWAS summary statistics are shown below the table, and “ic” indicates that the GWAS was performed using ImmunoChip. The “Condition” column indicates the experimental condition where the gene expression was quantified, with “M” and “T” indicating myeloid cells and CD4^+^ T cells, which were stimulated by either lipopolysaccharide (LPS) or phytohemagglutinin (PHA), respectively. The “#Instruments” column indicates the number of genetic instruments and the “Method” column shows the five MR methods that were performed. The “Causal estimate”, “SE”, and “P-value” columns indicate the estimated causal effects on disease risk per sd change in gene expression, the standard error, and P-value, respectively. Here the table shows the associations that were significant (P-value <0.05) in at least three out of all four MR methods, and significant ones are highlighted in bold. Causal associations with significant average pleiotropic effects (i.e. with significant intercept in the MR-Egger method) were excluded.
